# Neocortical and cerebellar malformations affect flurothyl-induced seizures in female C57BL/6J mice

**DOI:** 10.3389/fnins.2023.1271744

**Published:** 2023-11-02

**Authors:** Katherine M. Keever, Ying Li, Paige D. Womble, D. Gregory Sullens, Gonzalo H. Otazu, Joaquin N. Lugo, Raddy L. Ramos

**Affiliations:** ^1^Department of Biomedical Sciences, New York Institute of Technology College of Osteopathic Medicine, Old Westbury, NY, United States; ^2^Department of Psychology and Neuroscience, Baylor University, Waco, TX, United States

**Keywords:** neuronal migration, C57BL/6J mice, heterotopia, mouse models, epilepsy, cortical dysplasia, sex differences, cerebellum

## Abstract

Brain malformations cause cognitive disability and seizures in both human and animal models. Highly laminated structures such as the neocortex and cerebellum are vulnerable to malformation, affecting lamination and neuronal connectivity as well as causing heterotopia. The objective of the present study was to determine if sporadic neocortical and/or cerebellar malformations in C57BL/6J mice are correlated with reduced seizure threshold. The inhaled chemi-convulsant flurothyl was used to induce generalized, tonic-clonic seizures in male and female C57BL/6J mice, and the time to seizure onset was recorded as a functional correlate of brain excitability changes. Following seizures, mice were euthanized, and brains were extracted for histology. Cryosections of the neocortex and cerebellar vermis were stained and examined for the presence of molecular layer heterotopia as previously described in C57BL/6J mice. Over 60% of mice had neocortical and/or cerebellar heterotopia. No sex differences were observed in the prevalence of malformations. Significantly reduced seizure onset time was observed dependent on sex and the type of malformation present. These results raise important questions regarding the presence of malformations in C57BL/6J mice used in the study of brain development, epilepsy, and many other diseases of the nervous system.

## Introduction

Several parts of the central nervous system exhibit a highly laminated cytoarchitecture. For example, in both the cerebellum and neocortex, neuronal lamina are created by sequential rounds of cell division by neuronal progenitors followed by progressive migration of newly generated neurons into distinct layers (Angevine and Sidman, 1961; Altman, 1972). Aberrant neuronal proliferation can alter the size of the neocortex and cerebellum causing disorders such as microcephaly and cerebellar hypoplasia (Basson and Wingate, 2013; Accogli et al., 2021). Similarly, deficits in neuronal migration can disrupt lamination and cause heterotopia in these regions (Bahi-Buisson and Guerrini, 2013; Parrini et al., 2016).

Malformations of neocortical development due to altered neuronal migration are very often comorbid with seizures and epilepsy (Guerrini et al., 2003; Guerrini and Dobyns, 2014). Moreover, epilepsy associated with malformation of neocortical development is often refractory to anti-seizure medications (Harvey et al., 2008). For this reason, surgical interventions in refractory epilepsy due to altered neuronal migration often include resection of dysplastic or heterotopic tissue (Meroni et al., 2009; Cossu et al., 2018). Recently, a growing number of clinical studies as well as research using experimental animal models have also linked disorders of cerebellar development with seizures and epilepsy (reviewed in Streng and Krook-Magnuson, 2021; Rondi-Reig, 2022). Taken together, these data support the hypothesis that disorders of neuronal migration are characterized by changes in neuronal architecture as well as circuit rewiring that can lead to brain hyperexcitability.

Animal models with neocortical malformations consistently demonstrate a phenotype of spontaneous seizures and/or reduced seizure threshold (Lee et al., 1997; Gabel and LoTurco, 2002; Croquelois et al., 2009; Gilbert et al., 2014; reviewed in Wong and Roper, 2016). However, similar studies in models with cerebellar malformation are lacking. C57BL/6J inbred mice are a well-suited inbred strain to study the relationship between seizures and neocortical and/or cerebellar malformations. This strain has sporadic, but highly consistent malformations of the neocortex (Ramos et al., 2008) and cerebellum (Ramos et al., 2013). In the present report, we used the inhaled chemi-convulsant flurothyl to provoke seizures in male and female C57BL/6J mice and performed a detailed histological analysis of neocortical and cerebellar cytoarchitecture. We found that the presence of malformations significantly reduced the seizure threshold in female mice. Our results are discussed in the context of the use of C57BL/6J mice in studies of brain development, epilepsy, and other nervous system disorders.

## Methods

Male and female C57BL/6J mice aged between 12 and 16 weeks were used in the following study. Mice were purchased from The Jackson Laboratory and bred at Baylor University to produce male and female offspring. A total of 131 mice, 69 male and 62 female, were used in this study. Testing for both cohorts took place between postnatal days (PD) 60–70. The mice were group-housed in standard acrylic mouse cages with *ad libitum* access to both standard mouse chow and water, with no more than five mice being housed in a single cage. Testing occurred during the light cycle. The colony was maintained at 22°C on a 12 h light/12 h dark diurnal cycle. All procedures performed complied with the *Guide for the Care and Use of Laboratory Animals* from the National Institutes of Health and were approved by the Baylor University Institutional Animal Care and Use Committee.

The chemi-convulsant flurothyl was used to induce an acute seizure as previously described by our group (Holley and Lugo, 2016; Holley et al., 2018). Each seizure was induced under a standard fume hood inside a clear acrylic (29 × 16 × 15 cm) inhalation chamber. Specifically, the mice habituated to the room for 30 min prior to induction in their home cage and then were placed in a clean transfer cage. Each mouse was placed individually into an acrylic inhalation chamber wherein undiluted flurothyl (bis-2,2,2-trifluroethylether), obtained from Sigma-Aldrich (St. Louis, MO, USA, product number: 287571), was pumped into the chamber using an extended glass syringe (14.57 mm) and the Harvard Apparatus model 11Plus syringe pump at a rate of 30 μL per minute. Flurothyl was administered until the mouse exhibited a generalized seizure as described in the standard Racine scale (Racine, 1972) including bilateral tonic extension of the hindlimbs which was often followed by wild running. The time to seizure onset was recorded by two investigators who were blind to whether a mouse had malformation or not. High inter-rater reliability in seizure latency scoring was ensured during training sessions prior to data collection for this study. After a seizure was induced, the mouse was removed from the chamber, placed into an individual transfer cage, and monitored for 1 h to allow time for recovery.

Following seizures, the mice were euthanized and decapitated, and brains were placed in 4% paraformaldehyde for at least 24 h. Histological methods developed and validated to identify both cerebellar and neocortical malformations in C57BL/6J mice were used as previously described (Lipoff et al., 2011; Mangaru et al., 2013; Ramos et al., 2013, 2014, 2016; Van Dine et al., 2013a). Brains were placed in 30% sucrose for 48 h before undergoing sectioning on a cryostat. The forebrain and cerebellum were separated from one another. The entire forebrain was cut coronally and the entire cerebellar vermis was cut along the sagittal plane at a thickness of 45 μm, with free-floating sections placed in saline. Every other coronal section (~60 total sections) of the forebrain and ~15 sections of the cerebellar vermis were mounted on gelatin-coated slides and allowed to dry for at least 24 h. The slides were Nissl-stained and then cover-slipped. Brightfield microscopy at varying magnifications (4, 20, and/or 40X) was used to screen sections for cerebellar and neocortical malformations. Digital photomicroscopy was used to document malformations according to methods previously used to identify both cerebellar (Van Dine et al., 2013a,b, 2015; Ramos et al., 2015) and neocortical malformations (Ramos et al., 2008; Toia et al., 2017). Statistical analysis and creation of graphs were performed via GraphPad Prism version 9.4.0 with significance levels set at a *p*-value of <0.05. Seizure onset data are reported in seconds (s) ± the standard error of the mean (SEM).

## Results

**Figure 1:**
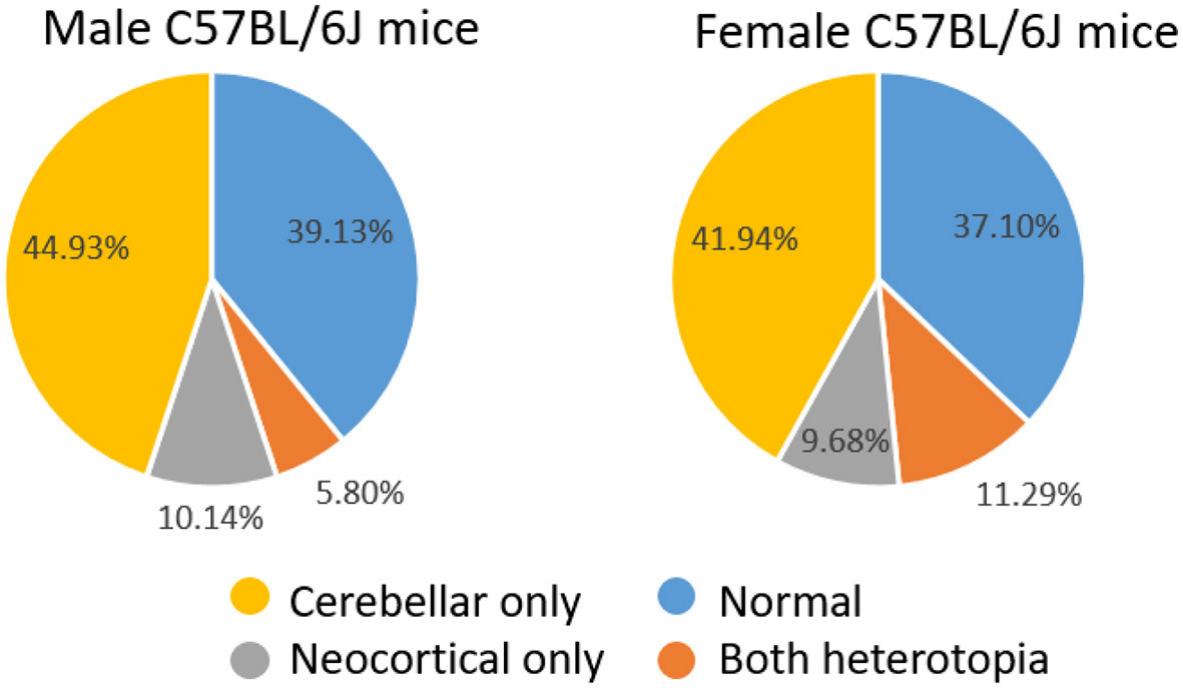
Distribution of male and female mice with normal brains or heterotopia in the neocortex and/or cerebellum.

We have previously described the prevalence and cytoarchitecture of neocortical and cerebellar heterotopia in C57BL/6J mice (Ramos et al., 2008; Toia et al., 2017). Consistent with our previous work, we found that a large proportion of mice had cerebellar and/or neocortical heterotopia. A total of 40 of 62 (64.5%) female mice and 42 of 69 (67.74%) male mice had either type of malformation. Chi-square analyses revealed no sex difference in the prevalence of having a malformation. As shown in Figure 1, among male mice, 31 (44.93%) had cerebellar heterotopia only, 7 (10.14%) had neocortical heterotopia only, and 4 (5.80%) had both types of malformation. Chi-square analyses indicated that the prevalence of cerebellar malformation alone is significantly greater than that of neocortical heterotopia alone or having both types of malformations (*X*^2^ = 29.91, *p* < 0.00001; Yates correction 25.88; *p* < 0.0001). As shown in Figure 1, among the female mice, 26 (41.94%) had cerebellar heterotopia only, 6 (9.68%) had neocortical heterotopia only, and 7 (11.29%) had both types of malformation. Chi-square analyses indicated that the prevalence of cerebellar malformation alone is significantly greater than that of neocortical heterotopia alone or having both types of malformations (*X*^2^ = 14.91, *p* < 0.0002; Yates correction 13.3786; *p* < 0.0003). Comparison between male and female mice based on the prevalence of having one type of malformation (e.g., cerebellar only) or having both types of malformations was non-significant.

**Figure 2:**
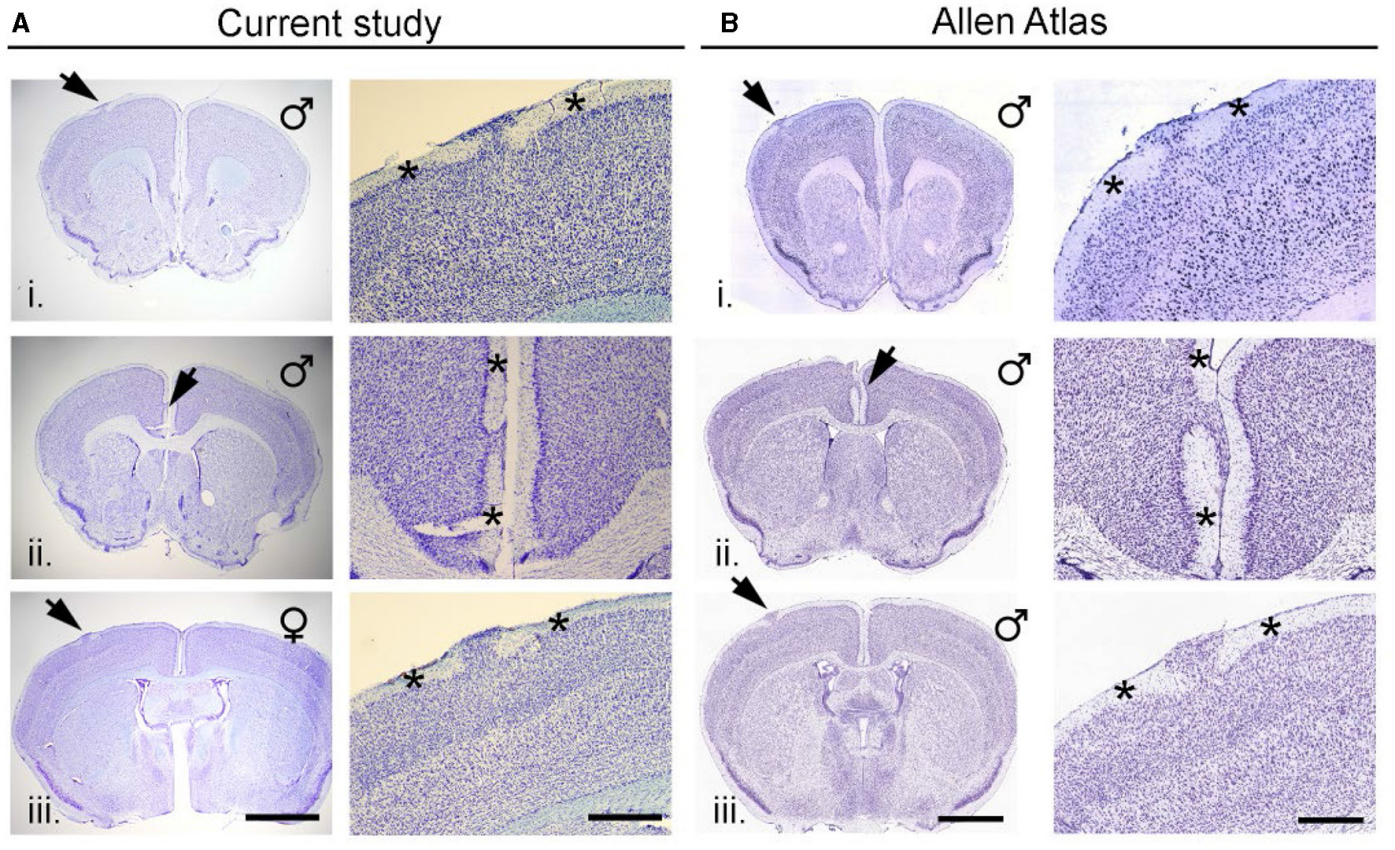
Neocortical heterotopia in C57BL/6J mice. **(A)** Three examples (i–iii) of neocortical heterotopia (arrows) from mice in the current study. Heterotopia could be in the dorsal neocortex (i and iii) or at the cortical midline (ii) and was characterized by an accumulation of neurons and glia in layer 1 which would otherwise have few scattered neurons therein. High magnification of heterotopia shown in left-side panels with asterisks indicate areas of intact layer I adjacent to heterotopia. **(B)** Three examples (i–iii) of neocortical heterotopia taken from the Allen Mouse Brain Atlas that are nearly identical in spatial/areal location at the dorsal neocortex (i and iii) or the cortical midline (ii) scalebar for **(A)** (left/right) = 1,047/400 μm; **(B)** (left/right) = 1,500/600 μm.

Figure 2A shows three examples of neocortical heterotopia (arrows) from mice in the current study. Heterotopia could be found in the dorsal neocortex (i, iii) or at the cortical midline (ii) and was characterized by an accumulation of neurons and glia in layer I, which would otherwise have few scattered cells therein. High magnification of heterotopia (Figure 2A, right-side panels) indicated areas of intact layer I adjacent to heterotopia (asterisks). As we described previously, heterotopia are never found in lateral (e.g., auditory cortex, perirhinal, or entorhinal) or posterior cortices (visual cortex). Figure 2B shows three examples (i-iii) of neocortical heterotopia taken from the Allen Mouse Brain Atlas that are nearly identical in spatial/areal location at the dorsal neocortex (i, iii) or the cortical midline (ii) to data found in our current study.

**Figure 3:**
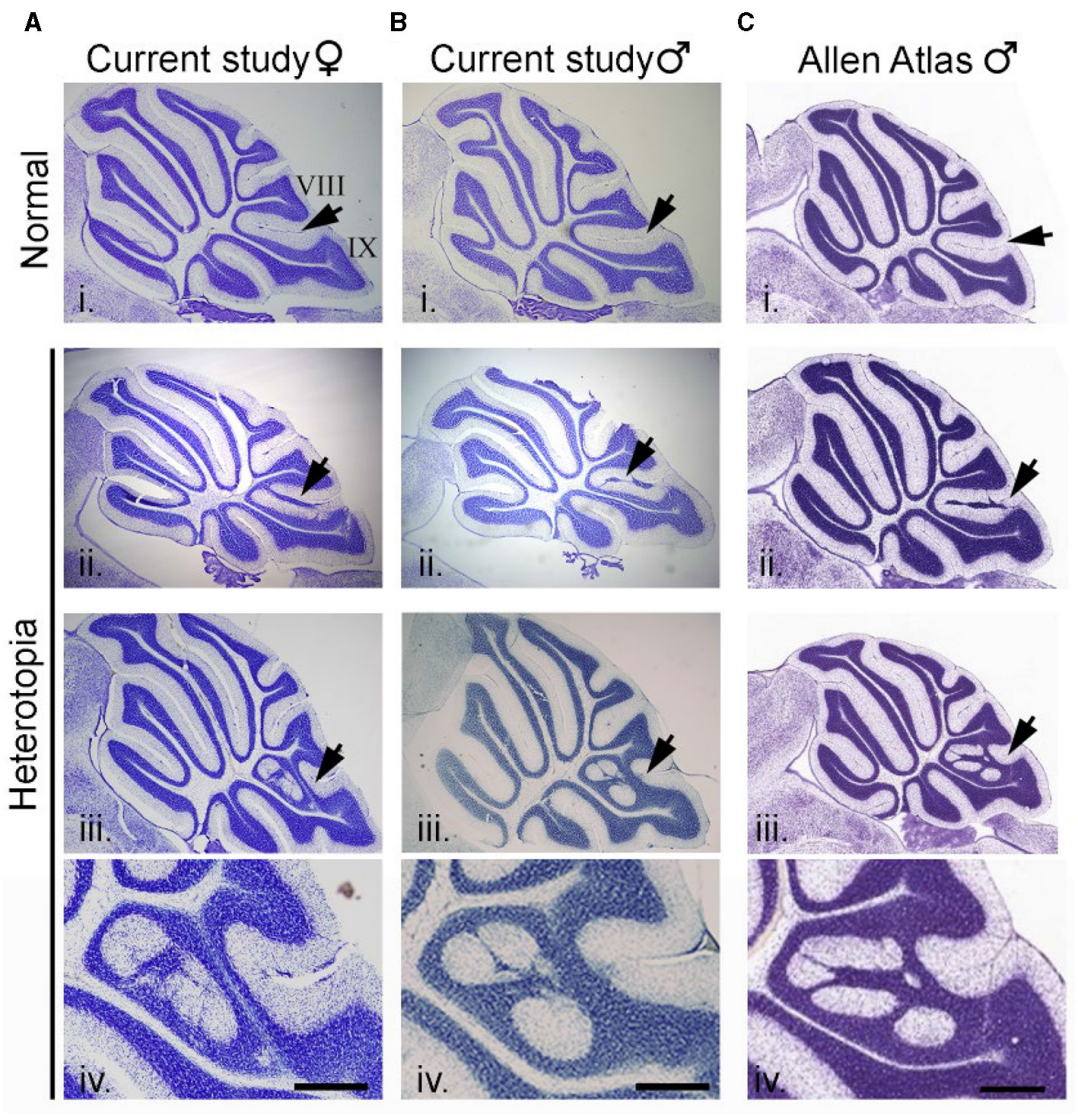
Cerebellar heterotopia in C57BL/6J mice. **(A)** Example of an intact cerebellum (i) and two examples of cerebellar heterotopia (ii and iii) in female mice from the current study. Arrows point to normal cytoarchitecture of lobules VIII and IX (i) or heterotopic neurons and glia in between lobules VIII and IX (ii and iii). **(B)** Example of intact cerebellum (i) and two examples of cerebellar heterotopia (ii and iii) from male mice from the current study. **(C)** Example of intact cerebellum (i) and two examples of cerebellar heterotopia (ii and iii) from male mice from the Allen Mouse Brain Atlas which are nearly identical to that from the current study. Higher magnification of examples in (iii) are shown in (iv). Scalebars for **(A, B)** = 1,000 and 333 μm (i–iii and iv, respectively). Scalebars for **(C)** = 1,047 and 350 μm (i–iii and iv, respectively).

Cerebellar heterotopia are found exclusively at the vermal midline and only between lobules VIII and IX. As previously described, cerebellar heterotopia are characterized by breaches of the pia between lobules VIII and IX. This results in the disorganization of Bergmann glia and ectopic granule cells and Purkinje cells that accumulate in the molecular layer (Van Dine et al., 2015). Figures 3A, B show two representative examples of heterotopia in male and female mice in the current study. As shown in Figure 3C, near-identical malformations of the cerebellum could be found in the C57BL/6J dataset of the Allen Mouse Brain Atlas.

### Time to seizure onset analyses

**Figure 4:**
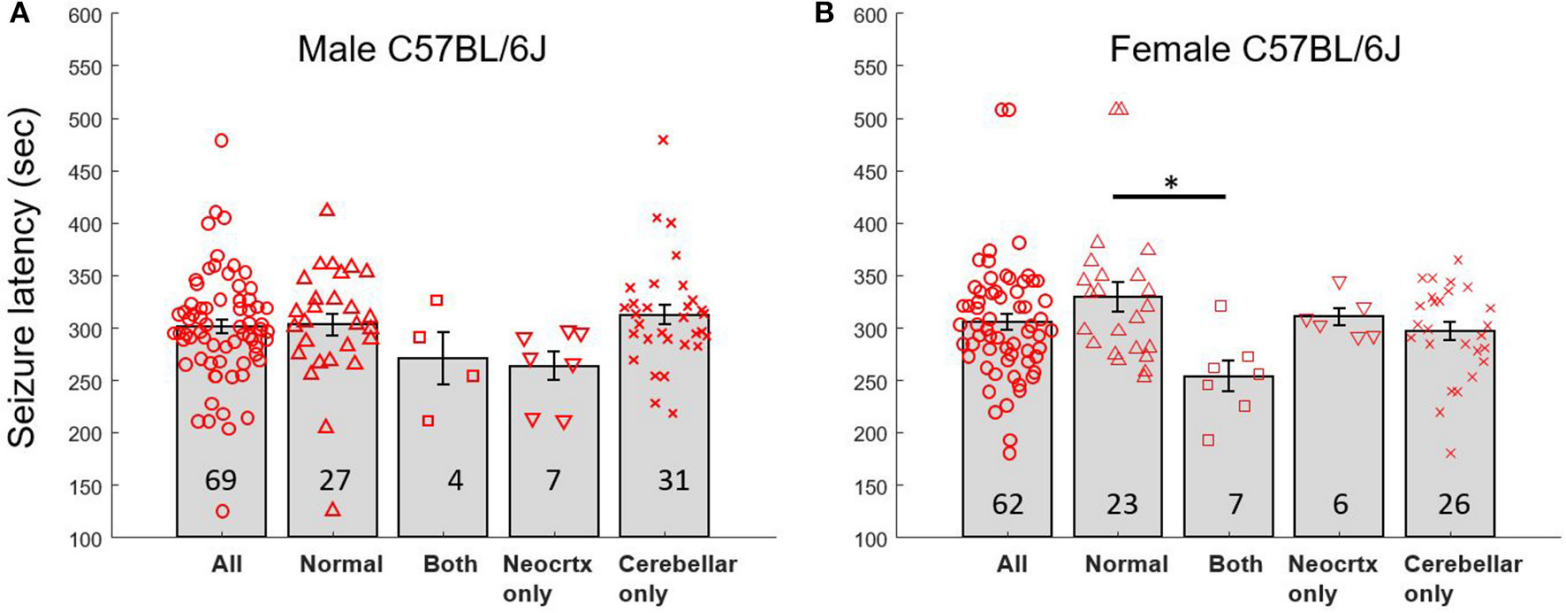
Seizure latency times for male and female mice with normal brains and heterotopia. **(A)** Latency to seizure (mean sec; error bars are SEM) for male C57BL/6J mice exhibiting a normal brain with no malformation, a brain with both types of malformation (Both), neocortical heterotopia only (Neocrtx only), or cerebellar heterotopia only. The numbers inside the bars reflect the number of mice in each group. **(B)** Latency to seizure (mean sec; error bars are SEM) for female C57BL/6J mice. Black bar and asterisk point to significant differences between selected groups. The numbers inside the bars reflect the number of mice in each group.

Our experimental approach was designed so that all mice experienced generalized seizures, and the time to seizure onset was used as a measure of brain excitability. In addition, we were blind to whether a mouse had a normal brain or exhibited cerebellar and/or neocortical malformations until histological analyses were performed. Given that we examined mice of both sexes and that mice could exhibit several malformation phenotypes (neocortical heterotopia only, cerebellar heterotopia only, heterotopia in both the neocortex and cerebellum), a number of different comparisons were performed among subgroups. Table 1 and Figures 4A, B display mean seizure latency times and SEM values for all relevant groups included in statistical comparisons.

**Table 1:** Average seizure latency in sec ± SEM for all relevant groups in the current study.

	**Male**	**Female**	**Total combined**
All	301.13 ± 6.39	305.44 ± 7.18	303.17 ± 4.77
Normal	302.90 ± 10.62	329.60 ± 14.12	315.18 ± 8.78
Neocortical only	263.60 ± 8.14	310.30 ± 13.92	285.15 ± 10.50
Cerebellar only	312.00 ± 9.24	296.80 ± 8.66	305.09 ± 6.42
Both	270.50 ± 24.67	253.90 ± 15.03	259.91 ± 12.61

A two-way ANOVA was first used to compare seizure onset time between mice incorporating both sex and brain phenotype as variables. This analysis revealed a significant main effect of brain phenotype (*p* < 0.02, *f* = 3.94) but no significant difference between sexes and no significant interaction. A one-way ANOVA was used to explore differences within each sex. This analysis revealed a significant main effect of brain phenotype among female mice (*p* < 0.02, *f* = 4.1). *Post-hoc* Tukey tests revealed that seizure latency times differed significantly (*p* < 0.02, *Q* = 4.60) between female mice with normal brains (329.6 s ± 14.12 SEM) and female mice with both types of heterotopia present (253.9 s ± 15.03 SEM). In contrast, comparisons between all other groups did not reveal any significant differences. A one-way ANOVA was used to explore differences in seizure latency times of male mice. No significant main effect of brain phenotype was observed.

The above comparisons using ANOVA emphasize potential differences between all different subgroups of mice that have malformation. However, more general comparisons are also valuable such as between mice with a normal brain vs. the combined group of mice with any kind of malformation. For example, a two-tailed *t*-test of seizure latency in all mice with a normal brain compared to all mice with any kind of heterotopia revealed significant differences (*p* < 0.05, *t* = 2.00). A two-tailed *t*-test of seizure latency between female mice with a normal brain compared to female mice with any kind of heterotopia also revealed significant differences (*p* < 0.009, *t* = 2.72). In contrast, a two-tailed *t*-test of seizure latency between male mice with a normal brain compared to male mice with any kind of heterotopia revealed no significant differences. Bonferroni corrections applied to the above *t*-tests change the significance level threshold to <0.0167. Applying this new threshold, the seizure latency of all mice with normal brains compared to all mice with any kind of heterotopia would no longer be significant. However, the seizure latency of female mice with normal brains compared to all female mice with any heterotopia would remain significant.

## Discussion

The present study provides important findings relevant to many areas of neuroscience research using mouse models. First, we replicated our previous work demonstrating that C57BL/6J mice do indeed exhibit cerebellar and/or neocortical malformations using a sample size of male and female mice larger than any previous study (Ramos et al., 2008; Van Dine et al., 2015). Analyses of malformation prevalence indicate that ~60% of male or female mice will have a malformation of the cerebellum, neocortex, or both regions similar to previous findings. Thus, recognizing that C57BL/6J mice will have malformations is important to the biomedical research community using these mice. For example, in the production of KO mice or other genetically engineered (GE) mice using the C57BL/6J background, investigators with an interest in developmental neuroscience should anticipate that KOs will have heterotopia which might confound the phenotype attributed to gene perturbation (Cuoco et al., 2018). We recently demonstrated that two popular genetic mouse models of autism on the C57BL/6J background exhibit neocortical and cerebellar heterotopia (Otazu et al., 2021) due to the C57BL/6J background. Moreover, there exists the possibility that gene KO might increase or decrease the prevalence of heterotopia or alter the size or distribution of malformations when these models are produced on the C57BL/6J background. Genetic mouse models of epilepsy are becoming more widely used as it is easier to produce GE mice for novel genes linked to epilepsy. It is expected that GE mice on the C57BL/6J background will also exhibit heterotopia, which may influence the seizure phenotype in these mice. Thus, our findings point to the need for histological identification of mice with/without malformations for subsequent analyses when using wild-type C57BL/6J mice or GE mice on this background.

Second, we show that female mice with both cerebellar and neocortical heterotopia have reduced seizure threshold in the flurothyl model. Female mice with normal brains were also different than mice with any kind of malformation when compared as a combined group. Surprisingly, female mice with only neocortical heterotopia did not exhibit changes in seizure onset although this group has a small sample size because of the relatively low prevalence of these malformations in C57BL/6J mice. In contrast to findings in female mice, male mice with either one or both types of malformations did not show any differences in seizure onset times. These findings point to sex differences in the influence of heterotopia on brain excitability. One limitation of our study is that we did not monitor the estrous cycle of our female mice, which could have introduced behavioral variability in our data. However, a recent report also using flurothyl reported no difference in seizure onset time between female mice in proestrus/estrus compared to those in diestrus (Echevarria-Cooper and Kearney, 2023). Thus, future studies could examine the possible interaction between gonadal sex hormones and brain malformations in female C57BL/6J mice by monitoring and testing mice at different stages of the estrous cycle or alternatively by performing ovariectomies and hormone replacement. A major limitation of the former approach is that the brain phenotype of mice is not known *a priori*, making it challenging to design a study with sufficient power.

Our results differ from a previous study with male C57BL/10J inbred mice, which have a greater prevalence of neocortical heterotopia and exhibit a lower seizure threshold to pentylenetetrazole (PTZ)-induced seizures (Gabel et al., 2013). While every experimental approach for seizure induction has its strengths and limitations, we used flurothyl in this study because of our extensive expertise with this approach (Lugo et al., 2014; Holley and Lugo, 2016; Holley et al., 2018; Binder et al., 2022). Moreover, flurothyl is much easier to use compared to methods requiring intraperitoneal injection, where mice often need multiple doses of chemi-convulsant to provoke a seizure or never exhibit a generalized seizure, complicating measures of latency. Nevertheless, another important area for future research should focus on the extent to which heterotopia affect seizure threshold depending on the method used to induce seizures (i.e., PTZ or kainic acid).

Finally, we show a role for cerebellar heterotopia in seizure onset time albeit only in female mice which also had neocortical heterotopia. Attention to the role of the cerebellum in seizures and epilepsy is growing in the clinical setting as well as in experimental studies with animal models. For example, there is a greater appreciation that the cerebellum can influence the physiology of neocortical (Popa et al., 2013; Watson et al., 2014; Tremblay et al., 2019) as well as subcortical structures such as the hippocampus via polysynaptic pathways (Yu and Krook-Magnuson, 2015; Bohne et al., 2019; Watson et al., 2019; Zeidler et al., 2020). Neocortical modulation of cerebellar physiology has also been demonstrated (Ros et al., 2009). Moreover, stimulation of the cerebellum has been shown to control seizures in mouse models of temporal lobe epilepsy (Krook-Magnuson et al., 2014; Streng and Krook-Magnuson, 2020, 2021; Streng et al., 2021; Stieve et al., 2022) and thalamic absence epilepsy (Kros et al., 2015a,b; Eelkman Rooda et al., 2021). Exactly how malformation of lobules VIII/IX of the posterior cerebellar vermis disrupts the function of the cerebellum and interconnected circuits in C57BL/6J mice to promote seizures remains unknown and is an important topic for future investigation.

There are also significant gaps in our knowledge regarding the anatomy and physiology of neocortical heterotopia, particularly with regard to afferent and efferent synaptic connections and the presence of heterotopic networks that can promote seizures. In other inbred strains with similar neocortical heterotopia (NXSM-D/Ei and NZB/BlNJ), postsynaptic potentials recorded by *in vitro* whole-cell patch-clamp revealed both glutamatergic and GABAergic synapses onto neurons in heterotopia (Gabel and LoTurco, 2001). Epileptiform activity was more readily evoked in cortical slices from these inbred models compared to those without heterotopia (Gabel and LoTurco, 2001, 2002). Using *in vivo* calcium imaging in an induced heterotopia model, neurons in heterotopia were shown to be functionally connected to other brain areas; however, epileptiform activity was not observed (Li et al., 2021). Thus, while physiological data are consistent with immunohistochemical and tracer studies (Ramos et al., 2014) showing diverse connectivity in neocortical heterotopia, the role of altered neuronal circuitry in heterotopia in seizure generation remains poorly understood. Note that physiological recordings *in vivo* or *in vitro* have yet to be performed in spontaneous neocortical heterotopia in C57BL/6J mice.

In conclusion, we demonstrate that sex and the presence of heterotopia interact to modulate flurothyl-induced seizure threshold in C57BL/6J mice. These data point to the need to consider how heterotopia can affect studies of epilepsy using C57BL/6J mice, which is the most widely used strain in neuroscience research.

## Data availability statement

The raw data supporting the conclusions of this article will be made available by the authors, without undue reservation.

## Ethics statement

The animal study was approved by Baylor University Institutional Animal Care and Use Committee. The study was conducted in accordance with the local legislation and institutional requirements.

## Author contributions

KK: Data curation, Formal analysis, Investigation, Writing—original draft. YL: Formal analysis, Investigation, Writing—review & editing. PW: Conceptualization, Formal analysis, Investigation, Methodology, Writing—review & editing. DS: Conceptualization, Investigation, Methodology, Writing—review & editing. GO: Data curation, Formal analysis, Investigation, Writing—review & editing. JL: Conceptualization, Funding acquisition, Investigation, Methodology, Resources, Supervision, Writing—original draft, Writing—review & editing. RR: Conceptualization, Investigation, Project administration, Resources, Writing—original draft, Writing—review & editing, Formal analysis.
